# Soft tissue and intraosseous pneumatosis secondary to diabetic foot ulcer: a severe case of emphysematous osteomyelitis

**DOI:** 10.1093/bjrcr/uaaf016

**Published:** 2025-03-18

**Authors:** Emmanuel Olayinka Sobamowo, Mirza Shaheer Baig, Sumantra Kumar, Nikhil Rasik Patel

**Affiliations:** Department of Radiology, Princess Royal University Hospital Orpington, London BR6 8ND, United Kingdom; Department of Radiology, Princess Royal University Hospital Orpington, London BR6 8ND, United Kingdom; Department of Radiology, Princess Royal University Hospital Orpington, London BR6 8ND, United Kingdom; Department of Radiology, Princess Royal University Hospital Orpington, London BR6 8ND, United Kingdom

**Keywords:** emphysematous osteomyelitis, osteomyelitis, diabetic foot, charcots foot, charcots, charcot, pumice, intraosseous gas, *Enterococcus faecium*

## Abstract

Emphysematous osteomyelitis (EO) is an uncommon but severe form of osteomyelitis that is characterized by gas formation within the bone. This case report highlights a case of particularly severe EO in an amputated foot, with key imaging findings across modalities emphasizing the diagnostic challenges and the importance of early detection. A 68-year-old male with a history of poorly controlled diabetes and a previous left third to fifth toe amputation for a non-healing ulcer presented to the emergency department with an infective picture and poorly controlled blood glucose levels. After clinical assessment, a focus of infection was found in the left foot and was subsequently assessed with plain radiography, MRI, and CT. The case highlighted the utility of each modality in such a complex presentation, including trabecular bony changes on the plain radiograph, soft tissue changes on MRI and confirmation of intraosseous pneumatosis on CT. This case highlights key imaging features of EO and underscores the need to use CT and MRI to guide timely surgical and medical management. This report adds to the limited literature on EO and presents a useful acronym of “LEAP” to describe key features when suspecting EO – lack of cortical destruction, extra-osseous soft tissue gas, associated comorbidities (diabetes, malignancy, etc), and pumice stone sign.

## Introduction

Emphysematous osteomyelitis (EO) is a rare infection caused by gas-forming bacteria. It primarily affects the bones and can involve the adjacent soft tissues and joints.^1^ Imaging features include intraosseous pneumatosis, for which differentials can also include trauma, fractures, and degenerative disease. Here, we report a particularly severe case of EO of the mid and forefoot in a 68-year-old male with a history of poorly controlled diabetes mellitus and foot ulceration with previous toe amputation. Very few cases of EO have been reported,^2^ particularly with CT and MR imaging findings, and we aim to highlight key clinical and imaging findings with a review of similar cases in the current literature.

## Case presentation

A 68-year-old male with a history of poorly controlled type 2 diabetes mellitus and associated complications including foot ulceration with previous osteomyelitis requiring amputation of the left third to fifth toe, renal disease with transplantation, retinopathy, hypertension, and anaemia presented to the emergency department with a 3-day history of new confusion, fever, reduced oral intake, and high blood glucose readings. He was seen by the diabetic nursing team prior to admission, who reported his left foot ulcer to be weeping and foul smelling. Inflammatory markers were significantly raised, so he was commenced on broad-spectrum antibiotics and intravenous (IV) fluids. A blood culture taken on admission was positive for the growth of *Enterococcus faecium* and *Klebsiella*, which were resistant to the initial antibiotics prescribed, and he was subsequently switched to IV meropenem and vancomycin. A podiatry tissue culture taken was also positive for growth of the same organisms. Additional issues such as hyperglycaemia with ketosis, renal function decline, and hyperkalaemia were also addressed in the same admission.

**Figure 1.**
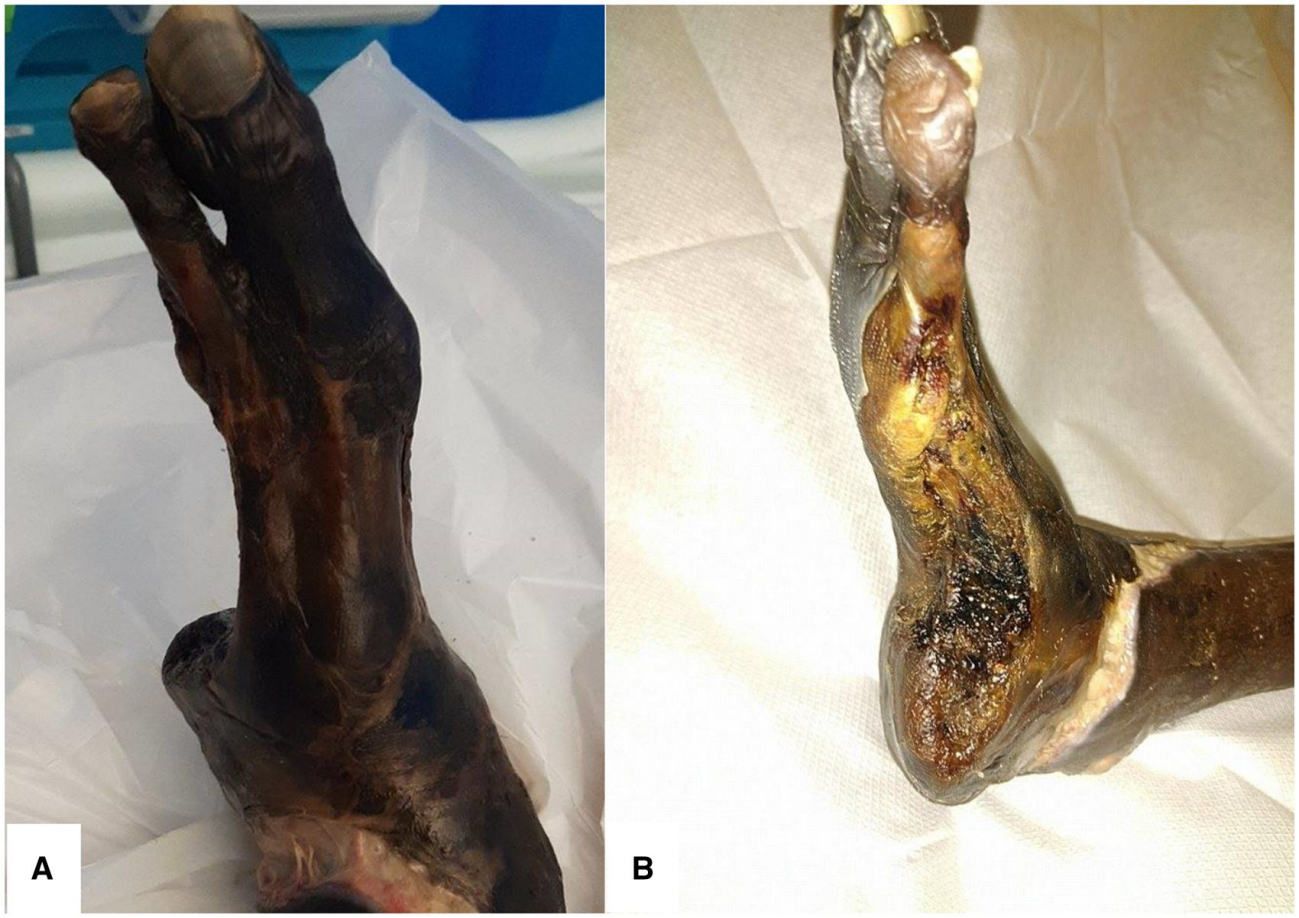
Initial photographs (A and B) demonstrate previous amputations of the third to fifth toes, the side view displays necrotic appearance of the superficial wound with peripheral blistering.

**Figure 2.**
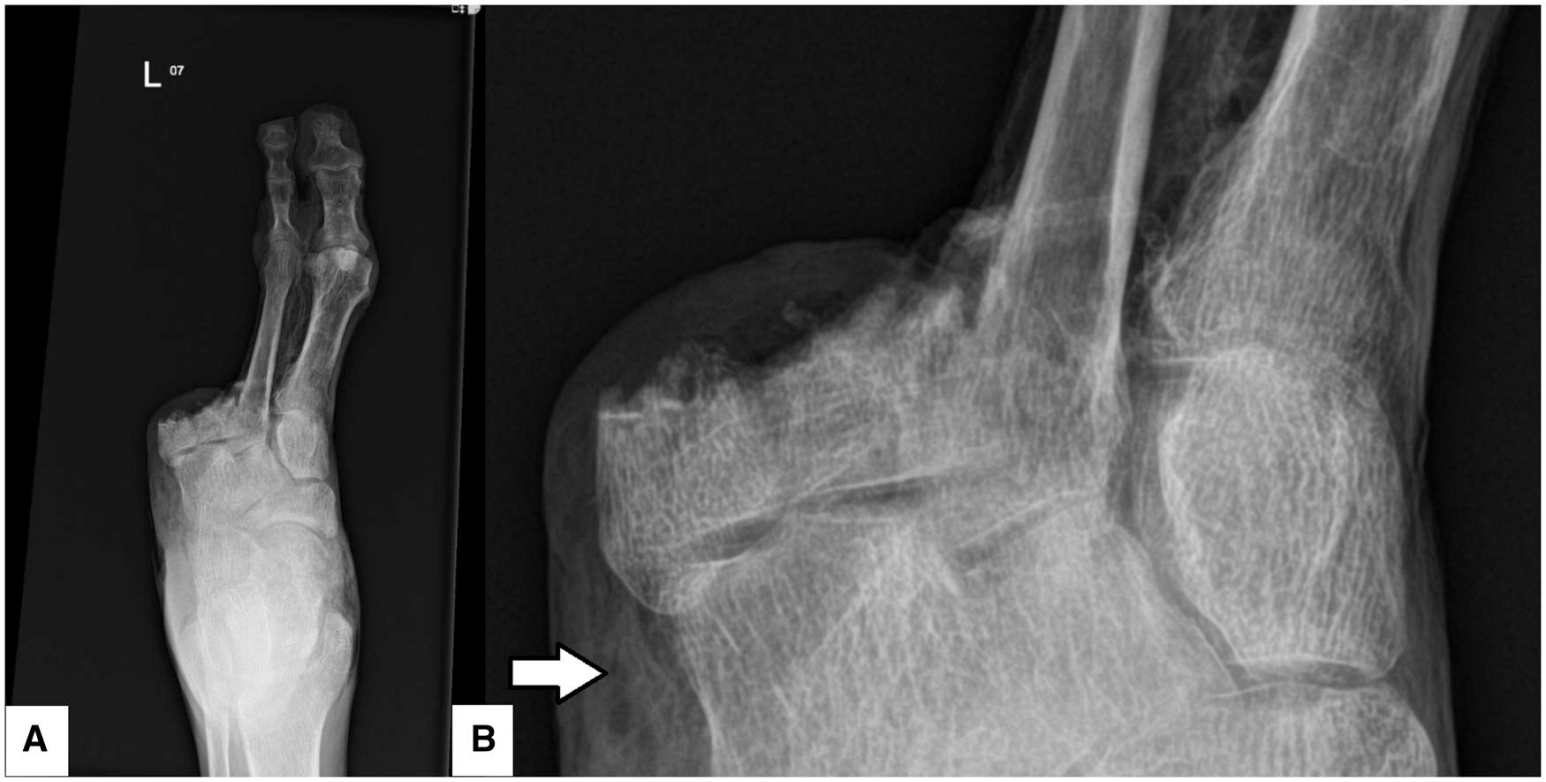
Left foot anteroposterior (AP) projection plain radiograph (A) and scaled-up view (B) demonstrating third to fifth toe amputation, soft tissue emphysema adjacent to the tarsal bones (arrow) and heterogenous trabecular bony pattern throughout, particularly within the remaining metatarsals.

Initial photographs (Figure 1) and a plain radiograph of the left foot demonstrated the previous third to fifth toe amputation, soft tissue emphysema, and heterogenous trabecular bone throughout (Figure 2).

**Figure 3.**
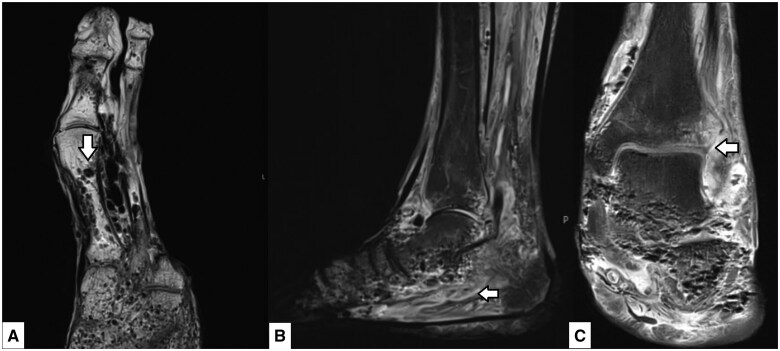
MRI of the left foot. Axial T1 weighted sequence slice (A) shows multiple rounded hypo-intensities/signal voids in the trabecular bone (arrow). Sagittal T2 STIR sequence slice (B) again demonstrates the same rounded hypo-intensities to a lesser extent as well as extensive soft tissue and intramuscular oedema (arrow). Coronal proton-density (PD) fat-saturated image slice (C) again demonstrated abnormality throughout the tarsal bone trabecula and surrounding soft tissues as well as the involvement of the tibiotalar joint (arrow).

An MR examination was carried out, which demonstrated extensive gas gangrenous soft tissue appearances with suspected tissue necrosis throughout the visualized mid and forefoot (Figure 3). Abnormal appearances of multiple rounded hypo-intensities in the bony distal tibia and midfoot were also noted as suspected intraosseous gas, and further evaluation with CT was advised.

**Figure 4.**
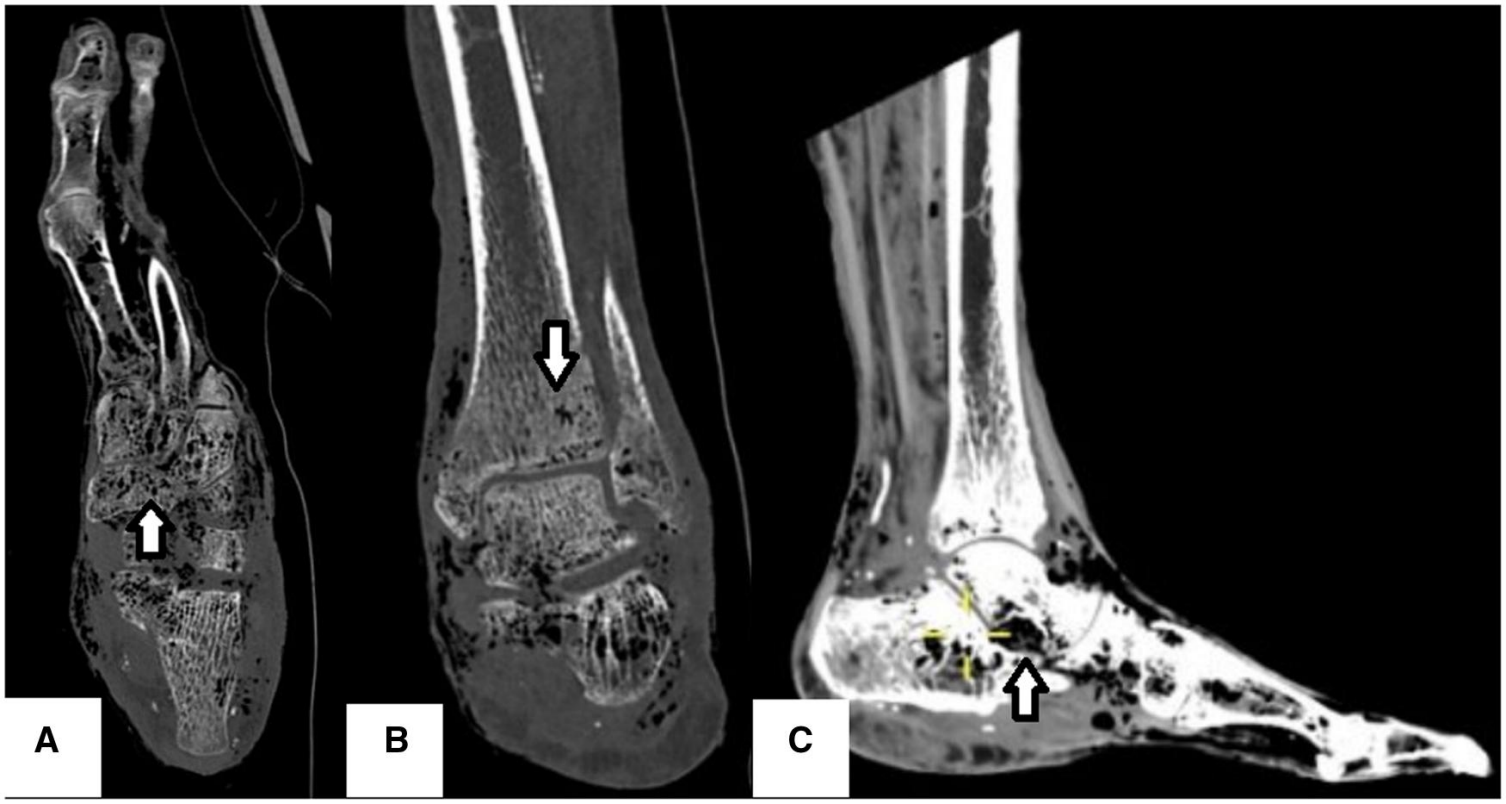
CT of the left foot. Axial (A) and coronal (B) slices view bony windows demonstrating locules of intraosseous gas demonstrated within the mid and forefoot, distal tibia, and fibular bones (arrows). Sagittal soft tissue window slice (C) displays a further locule of gas at the sinus tarsi and tracking along the soft tissues up to the mid to distal calf level (arrow).

A CT examination carried out 2 days later confirmed locules of intraosseous gas within the foot, distal tibia, and fibular suggestive of EO (Figure 4). Extensive bony involvement was noted and involved the distal phalanx of the first and second toes as well as tracking up along the soft tissues up to the mid to distal calf level with further locules of gas within the sinus tarsi.

Following the imaging findings, the case was discussed with the microbiology team with the decision to commence him on a course of long-term antibiotic therapy for 4 weeks on discharge via peripherally inserted central catheter (PICC line). Prior to discharge, his clinical status had improved with improving inflammatory markers as well satisfactory resolution of his concurrent renal and biochemical issues.

## Discussion

Emphysematous osteomyelitis is a rare aggressive infection mainly affecting the axial skeleton, with a high mortality of up to 32%.^3^ The characteristic feature of this condition is the presence of intraosseous gas which was first described as a CT finding in 1981 by Ram et al.^4^ Very few cases involving the extra-axial skeleton have been described; however, the presence of intraosseous gas in the extra-axial skeleton is highly indicative of EO, particularly once differential diagnoses such as trauma, that is, open fractures or penetrating wounds, open surgery or biopsy, osteonecrosis, degenerative disease, malignancy, and lymphangiomatosis of the bone, have been excluded. Although the majority of EO cases are located in the spine, intraosseous gas in this location is much more likely to be due to degenerative disease with features such as Schmorl’s nodes, vacuum disc phenomenon, gas-filled subchondral cysts, or intraosseous pneumatocysts.^1^ Of the few cases of extra-axial EO in the literature, the lower limb bones are the main locations reported, with the first published case involving the foot being described in 2014.^5^ The most common predisposing comorbidities, underpinned by the compromise of normal immune function, are diabetes mellitus, malignancy, and less commonly immunosuppressive therapy.^3^^,^^6^ Infection is usually spread via haematogenous dissemination; however, rare routes described include extension from intra-abdominal, skin, or soft tissue infection, as well as post spinal surgery.^6^ Gas-forming aerobic and anaerobic bacteria can cause infection; however, the most common causative organisms are anaerobes or members of the Enterobacteriaceae family.^3^ In addition, infection can be mono or poly microbial.

Clinically, traditional osteomyelitis and EO are similar in presentation with common features such as pain and fever; hence, imaging plays a key role in diagnosis.^7^ Radiography is usually good for the initial detection of intraosseous and soft tissue gas, with CT brilliant in the confirmation and further assessment of these findings.^6^ In our case, we also have MR images which, although not necessarily required for diagnosis, provide the added benefit of more detailed assessment of the soft tissues in particular. Soft tissue inflammation and emphysema adjacent to the affected bone were described in 79% of cases, as seen in our case, which additionally demonstrated tissue necrosis on MR.^1^ Small et al^1^ defined a novel sign termed the “pumice stone” sign, which described an intramedullary gas pattern consisting of clusters of more than 3 small foci of gas, irregular in size, measuring between 2 and 5 mm each. This sign was found in 96% of cases and identified in our case.^1^ Cortical destruction is seen in the majority of cases of classic osteomyelitis; however, it is much less common in EO (approximately 21% of cases).^1^

Emphysematous osteomyelitis carries a significantly high mortality and morbidity, and the radiologist’s role is important in diagnosis, assessing the extent of involvement, and prompting timely treatment. Due to the aggressive nature of this condition, intense antibiotic treatment of 4-6 weeks is the mainstay of treatment, with surgery required for potential complications like abscess or necrosis.^8^

## Learning points

- Although a very rare diagnosis, emphysematous osteomyelitis must be considered in the presence of intraosseous gas in the absence of any clear alternative differential diagnosis, particularly those that can cause a communication between bone and air, such as trauma and post-surgery.
- Emphysematous osteomyelitis and classic osteomyelitis may clinically present similarly, and imaging plays a key role in diagnosis. It is important to remember that traditional osteomyelitis tends to cause cortical destruction, which is much less common in emphysematous osteomyelitis.
- Features of emphysematous osteomyelitis to look out for, other than intraosseous gas, can be remembered by the acronym “LEAP”: lack of cortical destruction, extra-osseous soft tissue gas, associated comorbidities (such as diabetes and malignancy), and the pumice stone sign.
